# Impact of a Pharmacist-Driven Prothrombin Complex Concentrate Protocol on Time to Administration in Patients with Warfarin-associated Intracranial Hemorrhage

**DOI:** 10.5811/westjem.2018.6.37932

**Published:** 2018-08-13

**Authors:** Jessica L. Corio, Jonathan H. Sin, Bryan D. Hayes, Joshua N. Goldstein, Lanting Fuh

**Affiliations:** * Massachusetts General Hospital, Department of Pharmacy, Boston, Massachusetts; † Harvard Medical School, Department of Emergency Medicine, Boston, Massachusetts; ‡ Massachusetts General Hospital, Department of Emergency Medicine, Boston, Massachusetts

## Abstract

**Introduction:**

Advancements in the treatment of warfarin-associated intracranial hemorrhage (ICH) include the use of four-factor prothrombin complex concentrate (4F-PCC), which has demonstrated more rapid reversal of the international normalized ratio (INR) when compared with fresh frozen plasma. A pharmacist-driven protocol for 4F-PCC was implemented within our institution, which allows for pharmacist approval of 4F-PCC in patients diagnosed with warfarin-associated ICH and an INR ≥2. The pharmacist is responsible for determining the appropriate dose of 4F-PCC, preparation, bedside delivery, and order entry into the electronic medical record. Prior to implementation of the new protocol, the blood bank was responsible for 4F-PCC approval, dosing, product preparation, and arranging delivery with emergency department (ED) staff. The purpose of this study was to evaluate the impact of a pharmacist-driven protocol on time to 4F-PCC administration in warfarin-associated ICH.

**Methods:**

We performed a retrospective review of consecutive patients who received 4F-PCC in a single ED from September 2015 through February 2017. Patients ≥18 years old were eligible for inclusion based on three criteria: confirmed diagnosis of ICH; confirmed warfarin use; and INR ≥2. Secondary outcomes included dose of 4F-PCC in concordance with INR and weight-based dosing recommendations and hospital protocol, as well as concomitant intravenous vitamin K administration.

**Results:**

A total of 48 patients met inclusion criteria for the study with 24 patients in each protocol group. The median time to administration of 4F-PCC in the pharmacist-driven protocol group was 35 minutes (interquartile range [IQR] [25–62]; range, 11–133) compared with 70 minutes (IQR [34–89]; range, 14–244) in the pre-protocol group (p=0.034). We saw no differences for appropriate 4F-PCC dosing based on INR and patient weight between the two groups.

**Conclusion:**

Implementation of a pharmacist-driven protocol for 4F-PCC in the ED at our institution significantly reduced time to administration in patients presenting with warfarin-associated ICH.

## INTRODUCTION

Warfarin is a commonly prescribed oral anticoagulant indicated for prevention and treatment of venous thromboembolism and prevention of ischemic stroke in atrial fibrillation.^1^ One potential adverse effect of anticoagulation is bleeding, including intracranial hemorrhage (ICH). Until recently, warfarin-associated ICH in the United States was typically treated with fresh frozen plasma (FFP) and intravenous (IV) vitamin K.^2^ However, a four-factor prothrombin complex concentrate (4F-PCC) was approved by the U.S. Food and Drug Administration (FDA) in 2013 and has since become widely available for reversal of vitamin K antagonists, such as warfarin.^3^–^5^ The reversal effects of 4F-PCC occur through exogenous replacement of inactivated coagulation factors II, VII, IX, and X. The use of 4F-PCC has demonstrated a more rapid reversal of anticoagulation and the international normalized ratio (INR) compared with FFP.^6^,^7^

Clinical practice guidelines recommend prompt correction of the INR as soon as possible in patients with warfarin-associated ICH.^3^,^4^,^8^,^9^ Delays in administration of 4F-PCC for life-threatening bleeding have been recognized in several institutions, prompting review of hospital protocols. In a survey of United Kingdom (UK) stroke physicians, specific delays identified included time to hematology approval for 4F-PCC use, time to receive INR results, time to 4F-PCC delivery to the emergency department (ED), and time to infusion start.^10^ Solutions implemented within one healthcare system, based on survey results, included expanding approval privileges to include stroke physicians, purchasing point-of-care INR devices for bedside results, and moving 4F-PCC to be stored in the ED.

A second UK hospital observed a large delay in 4F-PCC administration with a median time of approximately five hours from initial presentation and almost two hours after vitamin K administration.^11^ Investigators also identified relocating 4F-PCC to the ED, incorporating point-of-care INR testing, and eliminating mandatory hematology consultations as future directions for reducing delays to administration. Bordeleau and colleagues evaluated administration delays of 4F-PCC prior to implementing a flowchart and new delivery process to improve communication between ED staff and the hospital blood bank.^12^ Use of the flowchart decreased time to obtain the product from the blood bank, and reduced time to 4F-PCC administration by almost half.

At our institution, the 4F-PCC was initially stored in the blood bank. To obtain the agent, ED clinicians contacted the blood bank for approval, and then ordered it via handwritten, paper slips that were delivered to the blood bank by pneumatic tube. The product was then prepared and delivered to the clinical nurse for infusion. To optimize 4F-PCC management, a new system was implemented in 2016 that led to product storage in the ED automated-dispensing cabinet and a revised protocol involving pharmacists physically present in the ED. Clinicians requesting 4F-PCC for warfarin-associated ICH contacted the ED pharmacist for dosing, preparation, bedside delivery, and order entry into the electronic medical record (EMR). The use of 4F-PCC for indications other than warfarin-associated ICH required approval from the on-call hematologist.

**Population Health Research Capsule**

What do we already know about this issue?

Many institutions have guidelines and restrictions for using four-factor prothrombin complex concentrate (4F-PCC) and may also require designated services to grant approval for using the reversal agent.

What was the research question?

We looked to evaluate implementation of a pharmacist-driven protocol on time to administration of 4F-PCC in warfarin-associated ICH.

What was the major finding of the study?

The pharmacist-driven protocol significantly reduced time to administration compared with the previous work-flow process.

How does this improve population health?

Incorporating emergency department (ED)pharmacists as an approval service for 4F-PCC and storing the reversal agent in the ED may reduce time to administration during episodes of life-threatening bleeding.

The purpose of this study was to assess the impact of the pharmacist-driven protocol on time to 4F-PCC administration in the ED for patients presenting with warfarin-associated ICH.

## METHODS

We conducted a single-center, retrospective review of consecutive patients issued 4F-PCC for warfarin-associated ICH. Patient characteristics collected included patient age, sex, type of ICH, and dose of 4F-PCC administered. Data points collected included ED registration time, initial INR, time INR was drawn and resulted, time ICH was confirmed on imaging, and documented time of 4F-PCC administration. We obtained institutional review board approval, and the need for informed consent was waived.

Our institution is a 1000-bed, Level I trauma and major regional referral center with more than 110,000 annual ED visits. During the study period, clinical pharmacists were physically present in the ED daily from 07:30 a.m. to midnight. During the overnight period, pharmacy services were provided from a separate, centralized location. The blood bank was staffed 24/7. Emergency medicine clinical pharmacy services were established in our ED prior to development of this protocol; therefore, no changes in the pharmacy-staffing model were required to support implementation of the pharmacist-driven 4F-PCC protocol.

We identified patients issued 4F-PCC between September 2015 and February 2017 from the hospital’s EMR and the blood bank data system. We chose parallel six-month pre- and post-implementation time periods for investigation. Patients treated between September 2015 and February 2016 were considered part of the pre-protocol group, and those treated between September 2016 and February 2017 were considered part of the pharmacist-driven protocol group. The new protocol was implemented in April 2016, but full education of clinicians was not yet complete and operational components were still being optimized. For this reason, we excluded this transitional period and included patients starting from September 2016 for the purposes of this analysis.

Patients were included in the analysis if they met the following criteria: ≥18 years of age; ICH confirmed on imaging; documented warfarin use; and initial INR ≥2 (Figure). Patients were excluded if they received more than one dose of 4F-PCC during the same hospitalization or if they received 4F-PCC under the pharmacist-driven protocol outside of ED clinical pharmacist coverage hours. To account for potential changes in staffing and blood bank workflow during the overnight time period, patients in the pre-protocol group were also excluded if they presented between midnight and 7:30 a.m. While hospital policy permitted 4F-PCC use for indications other than warfarin-associated ICH during both study periods, such use was rare and required extra levels of approval, introducing excess variability. As a result, we only analyzed those with warfarin-associated ICH for ease of analysis.

The primary outcome of the study was the amount of time from when the patient met criteria for 4F-PCC to the time of administration. The criteria required both an initial INR ≥2 and confirmation of ICH on imaging; the latter of the two recorded times was designated as the earliest time 4F-PCC was indicated for use. All time stamps were determined from the EMR, including the resulting time of the INR documented by the laboratory, and the final read time on neuroimaging results. For patients who were transferred to our facility, if both the patient’s INR and neuroimaging were already available from outside hospital records and used for 4F-PCC criteria, then the arrival time to our ED was designated as the starting time point. This starting time point was chosen since our ED clinicians receive the INR and neuroimaging information telephonically prior to the patient’s arrival. Once the patient arrived to the ED, they were already considered a 4F-PCC candidate. Our protocol’s intention was for the 4F-PCC procurement process to begin immediately upon arrival. However, if the transferred patient did not have both INR and neuroimaging results readily available and communicated to the clinicians, then the starting time point was pushed back until the patient was officially deemed a 4F-PCC candidate, after the missing information was resulted in our ED.

Secondary outcomes included dose of 4F-PCC in concordance with INR and weight-based FDA-label dosing recommendations and hospital protocol, as well as concomitant IV vitamin K administration. We also evaluated in-hospital mortality between the two protocol groups.

For our study purposes, appropriate dosing of 4F-PCC was determined based on the patient’s pre-treatment INR and recorded weight at the time of administration. Administered 4F-PCC doses exceeding five units per kilogram above or below the recorded weight were operationally defined as “inappropriate.” This was to account for potential differences in estimation of patient weight, in cases when an accurate weight was not easily attainable.

We considered that if our protocol shortened time by 20 minutes it would be deemed clinically relevant. A power calculation showed that we would need 32 patients, 16 in each arm, to detect this difference at the p <0.05 level. We analyzed the primary outcome using the Mann-Whitney *U* test. Baseline characteristics, secondary outcomes, and clinical outcomes were assessed using Student’s t-test, chi-square test, or Fisher’s exact test where appropriate. A *p*-value <0.05 was noted to be statistically significant.

## RESULTS

A total of 79 patients were issued 4F-PCC during the two six-month observational periods under each protocol. Overall, we included 48 patients in the study, with 24 patients in the pre-protocol group and 24 in the pharmacist-driven protocol group (Table 1). The median time to administration of 4F-PCC in the pre-protocol group was 70 minutes (interquartile range (IQR) = 34–89; range, 14–244) compared with 35 minutes (IQR = 25–62; range, 11–133) in the pharmacist-driven protocol group (p=0.034). There were no significant differences in dosing, based on pre-treatment INR and patient weight, between the pre-protocol group and the pharmacist-driven protocol group (p=0.174). All patients, with the exception of two in the pre-protocol group, received concomitant IV vitamin K, either at a referring hospital or in our ED upon diagnosis of warfarin-associated ICH (Table 2). In-hospital mortality occurred at comparable rates between the two study populations (p=1).

## DISCUSSION

Overall, we found that our change in protocol was associated with a 35-minute decrease in time to administration of 4F-PCC for warfarin-associated ICH in the ED. Numerous changes were made simultaneously, including the need for approval, the use of a pharmacist at the bedside, and the change in storage location leading to more rapid accessibility. The pharmacist also played an active role in dosing the reversal agent accordingly, based on the patient’s INR and weight, and served as a resource at the bedside for answering staff questions regarding administration and monitoring.

Prior to implementation of the pharmacist-driven protocol, 4F-PCC was purchased by and stored within the blood bank. Ownership of the product was transferred from the blood bank to the Department of Pharmacy in April 2016 and is now purchased under the department‘s budget. The protocol was developed in conjunction with the transfer of ownership.

Other institutions have performed similar analyses of systems changes. Through changing the approval process to include stroke physicians, implementing point-of-care INR testing, and moving a stock of 4F-PCC to the ED, one UK institution was able to decrease time to administration from a median of 127 minutes (IQR, 111–208) to 58 minutes (IQR, 50–91).^10^ A Canadian institution developed a new protocol to replace written orders sent to the blood bank for 4F-PCC with a verbal order from the ED attending, and designated a specific orderly to retrieve the prepared product from the blood bank, leading to a 40-minute improvement in time to administration.^12^ With our new protocol in place, the time to 4F-PCC administration improved by a median of 35 minutes, which is comparable to previous reports demonstrating an improved administration time of 30–58 minutes after protocol implementation.^10^–^12^

A number of factors can lead to delays in 4F-PCC administration in a real-world setting. Pre-intervention, numerous steps were required to obtain 4F-PCC, and it is not clear which steps required more or less time. Post-intervention, fewer steps were required, a dedicated pharmacist was readily available, and the primary storage location was a medication-dispensing cabinet within the ED. While both protocols required an approval service, the blood-bank fellow in the pre-protocol group and the pharmacist in the new protocol group, the pharmacist may already be actively involved in other facets of the patient’s care and can assess the patient earlier in their ED visit. However, it is unclear whether the presence of the pharmacist, the fact that fewer steps were necessary, or an unmeasured confounder led to the changes in administration times. One strength of our study is that the data represent a real-world setting of 4F-PCC administration, rather than the controlled environment and selected patients in a clinical trial.

There are no guidelines regarding optimal time to administration of anticoagulation reversal agents in relation to clinical outcomes and mortality. Though this study demonstrated a reduced time to administration, it was not powered to detect differences in clinical outcomes. Recent literature comparing 4F-PCC to FFP in the setting of warfarin-induced ICH has suggested faster reversal may be associated with a lower risk of hematoma expansion.^13^–^14^ While it is known 4F-PCC more rapidly reverses the INR in comparison with FFP, it remains unclear how soon 4F-PCC should be administered after the onset of warfarin-associated ICH and if there are any clinically significant benefits with faster reversal. Further study is needed to assess clinical measures and outcomes in earlier reversal of anticoagulation for warfarin-associated ICH and the optimal timeframe for 4F-PCC administration.

## LIMITATIONS

There are several limitations to address within the design of this study. First, this was a single-center, retrospective chart review. Second, patients were only included if 4F-PCC was requested during clinical ED pharmacist coverage hours (7:30 a.m. to midnight daily). As workflow processes differ overnight, we were unable to assess the impact on time to administration during this time period. Third, as this was a retrospective study, there may have been differences between the time of administration documented in the chart and the true time of administration. While neuroimaging results may have been discussed verbally prior to documenting the final read in the EMR, time stamps from the EMR were used to eliminate variation between cases. Fourth, as this was an observational study, it is possible the change in 4F-PCC administration time was due to an unmeasured confounder rather than the change in workflow. However, we are unaware of any other clinical process surrounding 4F-PCC administration that changed during this time frame. Fifth, given the small sample size, we were underpowered to evaluate any effect on clinical outcomes.

## CONCLUSION

Our study found the use of an ED pharmacist, in combination with 4F-PCC storage directly in the ED, was associated with a significant reduction in time to 4F-PCC administration after warfarin-associated ICH.

## Figures and Tables

**Figure:**
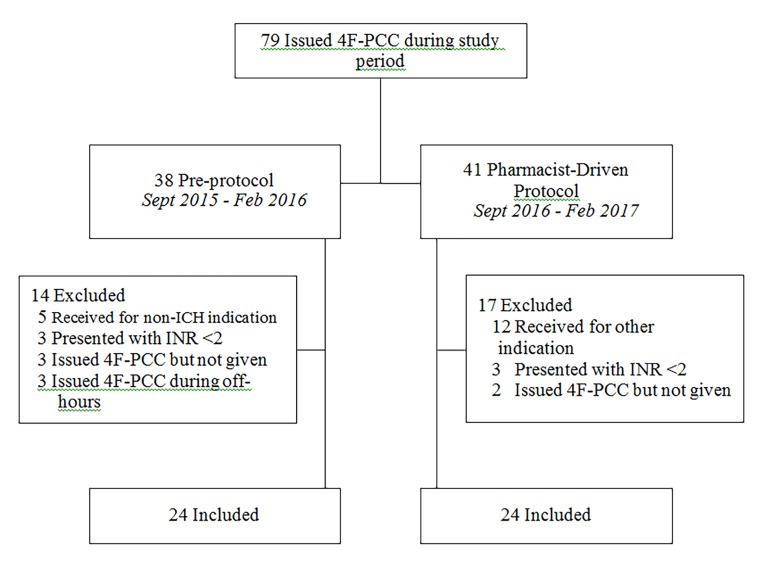
Inclusion and exclusion criteria. *4F-PCC,* four-factor prothrombin complex concentrate; *ICH*, intracranial hemorrhage *INR*, international normalized ratio.

**Table 1:** Patient demographics in study assessing impact of pharmacist-driven protocol for warfarin-associated intracranial hemorrhage.

Characteristic	Pre-protocol (n=24)	Pharmacist-driven protocol (n=24)	p-value
Age	76 (72–88)	83 (66–86)	0.59
Weight, kilograms	77.8 (60.5–87.5)	78.4 (65.2–83.4)	0.98
Gender, male	12 (50%)	11 (45.8%)	1.00
Transferred from outside hospital	15 (62.5%)	9 (37.5%)	0.15
Initial INR	2.3 (2.1–2.8)	2.7 (2.1–3.3)	0.35
2–3.9	22 (91.6%)	21 (87.5%)	1.00
4–6	1 (4.2%)	2 (8.3%)	1.00
>6	1 (4.2%)	1 (4.2%)	1.00
Location of ICH			
Subarachnoid	5 (20.8%)	2 (8.3%)	0.42
Intraventricular	2 (8.3%)	1 (4.2%)	1.00
Intraparenchymal	3 (12.5%)	8 (33.3%)	0.17
Subdural	9 (37.5%)	13 (54.2%)	0.39
Two or more sites	5 (20.8%)	0 (0%)	0.05

* All numbers are expressed as median (IQR) or n (%).

*INR*, international normalized ratio; *ICH*, intracranial hemorrhage.

**Table 2:** Study outcomes

	Pre-protocol (n=24)	Pharmacist-driven protocol (n=24)	p-value
Time to 4F-PCC administration, min	70 (34–89)	35 (25–62)	0.034
Appropriate 4F-PCC dosing‡			
Appropriate	20 (83.3%)	23 (95.8%)	0.174
Dose less than recommended	3 (12.5%)	1 (4.2%)	
Dose greater than recommended	1 (4.2%)	0 (0%)	
Concomitant vitamin K administration	22 (91.7%)	24 (100%)	0.244
In-hospital mortality	7 (29.2%)	7 (29.2%)	1

* All numbers are expressed as median (IQR) or n (%)

‡ Appropriate dosing based on international normalized ratio (INR) and weight-based FDA label dosing recommendations and hospital protocol.

*4F-PCC*, four-factor prothrombin complex concentrate.
